# Cellular Products with Anti-Inflammatory Properties for the Treatment of Cartilage Lesions

**DOI:** 10.3390/ijms27104316

**Published:** 2026-05-12

**Authors:** Polina A. Golubinskaya, Evgenii S. Ruchko, Alexandra N. Bogomazova, Artem V. Eremeev

**Affiliations:** 1 Lopukhin Federal Research and Clinical Center of Physical-Chemical Medicine, Federal Medical Biological Agency, Malaya Pirogovskaya 1a, Moscow 119435, Russia; 2 Center for Genetic Reprogramming and Gene Therapy, Lopukhin Federal Research and Clinical Center of Physical-Chemical Medicine of Federal Medical Biological Agency, Malaya Pirogovskaya 1a, Moscow 119435, Russia

**Keywords:** cellular therapy, articular cartilage, inflammation, osteoarthritis, macrophages, mesenchymal stem cells, chondrocytes

## Abstract

Most high-tech drugs and tissue engineering products based on human chondrocytes currently available on the market are aimed at restoring traumatic damage to cartilage tissue. However, in the presence of inflammation, their regenerative potential is significantly reduced, which limits their use in patients with osteoarthritis—one of the most common degenerative and inflammatory joint pathologies. The central element of the pathogenesis of osteoarthritis is inflammation—not classical acute inflammation, but rather chronic low-grade inflammation, primarily mediated by mechanisms of the innate immune response. Therefore, a key challenge is to enhance the anti-inflammatory effectiveness of cell-based drugs to broaden their indications to include degenerative diseases such as osteoarthritis and arthrosis. In recent years, cell-based drugs using stem cells, including mesenchymal stem cells (MSCs), hematopoietic stem cells (HSCs), and stromal vascular fraction (SVF) cells, have been actively studied. Despite their confirmed safety in inflammatory processes, meta-analyses of clinical trials show limited effectiveness in improving symptoms and MRI data in the treatment of osteoarthritis. A promising direction appears to be the development of combined cell-based drugs that combine MSCs with M2-polarized macrophages; however, data on their clinical effectiveness are still insufficient. This review explores key cellular effectors of inflammation and its molecular mechanisms, potential strategies for creating tissue engineering products that possess not only regenerative but also pronounced anti-inflammatory effects. The development of such products will expand their application in the treatment of inflammatory-degenerative joint diseases.

## 1. Introduction

Osteoarthritis (OA) is a chronic degenerative-inflammatory joint disease characterized by the progressive destruction of cartilage tissue [1]. According to literary sources, approximately 7.6% of the adult population worldwide suffers from OA [2], and by 2050, an increase in prevalence of 60–100% is projected [3].

The inflammatory process in OA is the result of a complex network of intercellular interactions, involving both resident joint cells and infiltrating immune cells [4,5,6]. Modern science portrays osteoarthritis as a complex inflammatory disease of the entire joint, rooted in dysfunction of the innate immune system stimulated by mechanical and metabolic cues [4,7,8,9]. Despite the diversity of molecular mechanisms involved, pro-inflammatory signaling pathways mediated by IL-1 and TNF-α remain the most clinically relevant as they play a central role in extracellular matrix degradation and activation of catabolic processes [10]. The principal pathways include not only classical cytokines and their signaling pathways (NF-κB, MAPK, JAK/STAT) but also fundamentally new mechanisms: inflammaging mediated by the accumulation of senescent cells with a senescence-associated secretory phenotype (SASP) [11], intercellular transfer of mitochondria enhancing inflammation [12], and regulation of programmed cell death (pyroptosis, ferroptosis) through non-coding RNAs and epigenetic modifications [8,13].

Importantly, the relative contribution of these pathways may vary depending on the patient-specific disease phenotype, underscoring the heterogeneity of OA and the need for personalized therapeutic strategies. Immune cells, particularly macrophages, are key orchestrators of this process [4,14,15], while the bidirectional “cartilage–bone” axis facilitates disease progression throughout the joint [16]. Recent studies have significantly expanded the understanding of the molecular and cellular foundations of OA pathogenesis, revealing numerous potential targets for pharmacological intervention.

The conservative treatment for OA primarily focuses on pain management rather than disease modification and reduction. Severe cases of OA are predominantly treated only by surgical methods [17]. Arthroscopic debridement, bone marrow stimulation to enhance chondrogenesis through microfracture of the subchondral bone, osteochondral grafting, and total joint arthroplasty are the most common surgical interventions. Arthroplasty is a widely used orthopedic technique for alleviating pain, increasing mobility, and improving joint function in OA [18]. However, each arthroplasty carries the risk of postoperative infections, chronic pain, and the need for revision surgery. For instance, a systematic review of prospective studies involving patients who underwent total knee arthroplasty showed unfavorable long-term outcomes in 10–30% of cases post-surgery [19]. All the above highlights the need to explore new therapeutic avenues for treating OA, including by combining immunomodulatory strategies and regenerative medicine [20]. Such approaches aim not only to alleviate the symptoms of OA but also to target the core immune mechanisms driving disease progression.

Regenerative medicine techniques offer an alternative therapeutic pathway for traumatic cartilage injuries [21]. These approaches, such as autologous chondrocyte implantation (ACI), mosaicplasty, and MSC transplantation/injection, are less invasive than conventional surgical approaches and target the underlying cause of the pathology.

ACI was the first cell-based replacement therapy strategy. One of the disadvantages of this approach is that chondrocytes cultured in a monolayer tend to dedifferentiate. This means they lose their specific phenotype and begin synthesizing type I collagen, which is more characteristic of fibrous cartilage, rather than type II collagen, typical of hyaline cartilage [22]. This process is accompanied by decreased expression of COL2A1 and increased COL1A1, ultimately leading to the formation of fibrocartilage rather than functional hyaline tissue [23,24].

Although regenerative approaches such as ACI were initially developed for the treatment of focal traumatic cartilage defects, their efficacy in osteoarthritis is limited by the inflammatory microenvironment. In addition, chondrocytes derived from osteoarthritic cartilage retain a pro-inflammatory phenotype, which further compromises their regenerative potential [25]. Consequently, the search for other approaches and cell sources for OA therapy continues. For instance, research by the M. G. Khotin group focuses on generating chondrocyte-like cells from fibroblasts [26]. Currently, this cell source is far from clinical application, as the protocols for differentiating fibroblasts into a chondrocytic lineage are insufficiently refined, and the resulting cells possess limited proliferative capacity and extracellular matrix (ECM) synthesis capability [26]. Therefore, successful cartilage regeneration in osteoarthritis requires not only cell replacement but also modulation of the local microenvironment. In this context, mesenchymal stem cells and anti-inflammatory M2 macrophages are considered key components for establishing a regenerative niche and maintaining a stable chondrogenic phenotype [27,28,29].

An alternative source of chondrocytes for transplantation is differentiated derivatives of induced pluripotent stem cells (iPSCs). It has been shown that chondrocytes derived from iPSCs are juvenile and differ from “adult” cells by accelerated proliferation and resistance to the cytokine IL-1β [30,31]. Currently, differentiated iPSCs derivatives are actively studied as promising material for the regenerative medicine of musculoskeletal lesions [32,33]. However, despite the promise, this approach has limitations. One limitation is the epigenetic memory of iPSCs: it is believed that despite dedifferentiation, they can retain expression features of the original somatic cell. In this case, using cells from patients with arthritis to obtain iPSCs and their subsequent differentiation into chondrocytes may be impractical due to the risk of preserving pathological epigenetic markers [34]. The use of MHC-matched allogeneic cells may be free of such risks [35]. Furthermore, the use of genetically edited hypoimmune iPSCs seems promising, which potentially solves the problem of autologous cell deficiency or the impossibility of taking a hyaline cartilage biopsy [35].

Thus, in cases of traumatic joint injuries, there are options for replacement therapy; however, each of these approaches has its own limitations and drawbacks. Yet they all demonstrate low efficacy in OA due to the formation of a pro-inflammatory microenvironment in the joint. It is known that OA is characterized by anatomical and physiological changes in joint tissues, including cartilage degradation, subchondral bone remodeling, and osteophyte formation, which lead to symptoms such as pain, swelling, and reduced mobility [36,37]. In addition to cartilage degradation, patients with OA exhibit pathological changes in the menisci [20] and synovial membrane [38]. These processes, worsening over time, create a persistent pro-inflammatory microenvironment in the joint. Importantly, spatial heterogeneity within joint tissues further contributes to variability in therapeutic outcomes. Articular cartilage exhibits a pronounced zonal organization, with each layer possessing distinct structural and functional properties that determine its regenerative capacity. Moreover, the affected joint displays tissue-level heterogeneity, including regions of hypo- and hypertrophy, subchondral bone remodeling, and ectopic osteoinduction, which collectively necessitate differentiated and site-specific therapeutic approaches [39].

OA is characterized by elevated levels of pro-inflammatory cytokines (such as IL-1β and TNF-α) and high activity of matrix metalloproteinases (MMPs). Consequently, even with the introduction of new cells, the pro-inflammatory environment can disrupt their survival and function [40]. In this regard, the scientific community is discussing the prospect of using tissue-engineered constructs containing an anti-inflammatory component—for example, macrophages and/or MSCs.

Macrophages, central regulators of innate immunity, are present in synovium and play a key role in OA progression by driving inflammation [14,41]. A defining characteristic of macrophages is their plasticity—the capacity to alter their phenotype and function in response to environmental cues. During immune responses, they can polarize into classically activated (M1, pro-inflammatory) or alternatively activated (M2, anti-inflammatory) states [42]. Although this M1/M2 classification is a simplification, as macrophages in vivo exist along a continuum of states depending on microenvironmental signals [43], it remains a useful conceptual framework. In this review, it is applied to highlight the role of anti-inflammatory macrophages in combination with mesenchymal stem cells. As M2 macrophages are crucial for tissue repair, shifting the polarization balance towards the M2 phenotype at the injury site could facilitate the repair of damaged articular cartilage.

The therapeutic potential of MSCs in OA stems less from their ability to differentiate into chondrocytes and more from their potent paracrine activity, which can inhibit chondrocyte apoptosis and attenuate overall tissue degeneration [36]. Moreover, MSCs exert immunomodulatory effects by secreting factors that suppress the activation of T-cells and neutrophils [37]. Critically, MSCs can directly modulate macrophage polarization, targeting a key cellular driver of advanced OA pathogenesis [14,38].

A critical consideration in designing tissue engineering strategies is the selection of cell types endowed with combined anti-inflammatory, immunomodulatory, and regenerative capacities. Despite the high therapeutic potential of mesenchymal stem cells, macrophages, and induced pluripotent stem cells, their clinical translation remains limited by several factors. These include safety concerns, such as the risk of residual undifferentiated cells and tumorigenicity in iPSC-derived products [44] phenotypic instability and heterogeneity of MSC [45], challenges in scalable manufacturing, and limited availability of source material, particularly monocytes for generating M2 macrophages from autologous sources [46]. iPSC-derived cells, including hypoimmunogenic lines, represent a promising alternative; however, they require rigorous safety evaluation. At the same time, advances in differentiation protocols, quality control systems, and scalable manufacturing platforms are progressively addressing these challenges.

Overall, these limitations underscore the need for multimodal therapeutic strategies combining immunomodulatory and regenerative approaches, with tissue-engineered constructs integrating MSCs, macrophages, and iPSC-derived cells emerging as a promising direction for improving treatment outcomes in OA.

## 2. Molecular and Cellular Mechanisms of Cartilage Inflammation in Osteoarthritis

Chondrocytes are the main type of cells in mature cartilage tissue. Under normal conditions, they maintain the homeostasis of the ECM by synthesizing type II collagen and aggrecan, characteristic of hyaline cartilage, and they exhibit low metabolic activity. In the presence of mechanical stress and pro-inflammatory mediators, the phenotype of chondrocytes changes [47]. They become not only targets but also active participants in inflammation, producing pro-inflammatory cytokines (IL-1β, TNF-α, IL-6), chemokines, nitric oxide (NO), and matrix metalloproteinases (MMP-1, -3, -13, ADAMTS-4, -5), which initiate the degradation of ECM [16,48]. Activated chondrocytes lose their differentiation, switching from the production of type II collagen to types I and III collagens (fibrosis) and type X collagen (a marker of hypertrophy) [49].

**Figure 1:**
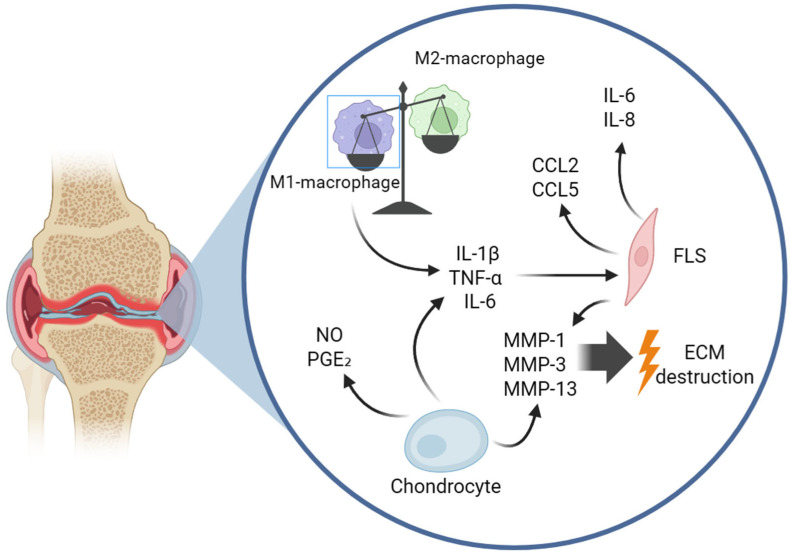
Part of the mechanism of molecular-cellular interaction in the pathogenesis of osteoarthritis. Abbreviations: FLS—fibroblast-like synoviocytes, NO—nitric oxide, MMPs—matrix metalloproteinases, PGE_2_—Prostaglandin E2. Figure created by the authors. Created in BioRender. Голубинская, П. (2026) https://BioRender.com/l72lrvq.

Synovitis is an integral component of the pathogenesis of OA, detected in both early and late stages of the disease [7,50,51]. The cells of the inflamed synovial membrane play a central role in the production of pro-inflammatory factors. In OA, fibroblast-like synoviocytes (FLS) become activated, hyperproliferate, and acquire an aggressive phenotype, secreting cytokines (IL-6, IL-8), chemokines (CCL2, CCL5), and MMPs (MMP-1, -3, -13) [9]. Modern studies reveal heterogeneity among FLS; for instance, there are reparative CILP+ fibroblasts and pro-inflammatory/pro-fibrotic POSTN+ fibroblasts associated with areas of cartilage damage [52]. The most numerous population of immune cells in the synovium are macrophages, and their polarization defines the balance between inflammation and repair [53]. M1 macrophages (classically activated) produce pro-inflammatory cytokines (IL-1β, TNF-α, IL-6, IL-12) and dominate in OA, correlating with disease severity and pain [5,54,55]. M2 macrophages (alternatively activated) produce anti-inflammatory cytokines (IL-10, TGF-β) and promote repair [56,57]. The imbalance toward the M1 phenotype is a key event, while its correction (repolarization to M2) represents a promising therapeutic strategy [15,58]. In the synovium of OA, mast cells (which promote angiogenesis and pain through tryptase) [59], neutrophils (whose elastase activates MMP-13) [60], and dendritic cells (activated via TLRs and promoting the differentiation of Th17 cells, thereby enhancing inflammation) [52,61] are also found. Although neutrophils are not as numerous as in rheumatoid arthritis, their presence correlates with the severity of OA [8,62] (Figure 1).

Remodeling of the subchondral bone is an integral part of OA pathogenesis. In the early stages, bone resorption predominates due to osteoclast activity, leading to thinning of the bone plate. In the later stages, sclerosis develops—an abnormal thickening of the bone due to the activity of osteoblasts that produce defective type I collagen [53]. This process is closely linked to inflammation: pro-inflammatory cytokines (IL-1β, TNF-α) stimulate the expression of RANKL on osteoblasts, activating osteoclastogenesis [63]. The “cartilage–bone” axis is bidirectional: factors from the remodeled bone (VEGF, TGF-β) can penetrate the cartilage, stimulating its degradation and angiogenesis, while cytokines from the cartilage in turn affect the bone [4,12,53].

Disruption of this axis involves alterations in bone metabolism, enhanced angiogenesis, and changes in mechanical properties, all of which contribute to cartilage degeneration. Current therapeutic strategies aim to modulate these processes through several key mechanisms: inhibition of osteoclast-mediated bone resorption to restore subchondral microarchitecture, suppression of aberrant angiogenesis (e.g., targeting VEGF signaling) to limit vascular invasion and cartilage degradation, and modulation of TGF-β signaling in subchondral bone to prevent pathological bone formation and subsequent cartilage damage [64]. Additionally, biomechanical interventions that reduce abnormal joint loading can further stabilize the cartilage–bone unit and slow disease progression [65].

The interaction between cells and the maintenance of inflammation is coordinated by a complex network of signaling molecules [52,58]. Primarily, these are pro-inflammatory cytokines such as IL-1β, TNF-α, and IL-6. IL-1β is considered the main regulator of catabolism in OA. It is synthesized by macrophages and chondrocytes as an inactive precursor, pro-IL-1β, which is cleaved by caspase-1 (within the inflammasome) into its active form [10,66]. By binding to the IL-1RI receptor, IL-1β initiates signaling pathways (NF-κB, MAPK), resulting in: (1) induction of MMP and ADAMTS; (2) suppression of type II collagen synthesis; (3) stimulation of the production of other mediators (IL-6, IL-8, NO, PGE_2_); (4) induction of apoptosis in chondrocytes [10,48,67]. TNF-α acts synergistically with IL-1β, activating similar pathways through TNFR1/TNFR2 receptors. It promotes matrix degradation, inhibits its synthesis, and participates in the induction of apoptosis [68,69]. Polymorphisms in the TNF-α genes are associated with a predisposition to OA. IL-6 is a pleiotropic cytokine, and its levels correlate with pain and joint dysfunction [70]. Trans-signaling of IL-6 plays a key role in the chronicity of inflammation, wherein the cytokine binds to a soluble receptor (sIL-6R), and this complex activates cells that lack the membrane-bound receptor [71]. IL-6 promotes synovial hyperplasia, immune cell infiltration, and MMP production. Despite successes in rheumatology, the use of IL-1β (anakinra) and TNF-α inhibitors (adalimumab) in OA has yielded modest clinical results, likely due to the heterogeneity of the disease and the absence of a single driver [72]. Emerging evidence suggests that stratification of patients, particularly the identification of an inflammatory OA phenotype, may improve therapeutic efficacy by enabling more targeted treatment approaches [73].

In addition to cytokines, other molecules play a role in the development of OA. Chemokines direct the migration of leukocytes to the joint. CCL2 (MCP-1) is the main chemoattractant for monocytes/macrophages, and its blockade reduces the severity of OA in models [74]. CCL3, CCL4, and CCL5 (RANTES) attract T cells and monocytes [75]. CXCL8 (IL-8) attracts neutrophils and stimulates chondrocyte hypertrophy [76]. CXCL12 (SDF-1) interacts with CXCR4, facilitating cell migration and possibly pathological calcification [77].

Obesity is a risk factor for the development of OA, acting not only through mechanical load but also through systemic inflammation induced by adipose tissue dysfunction. Adipokines produced by adipose tissue play a role in metabolic inflammation [6,78]. Leptin levels correlate with BMI and OA severity. Leptin has pro-inflammatory effects, stimulating the production of IL-6, IL-8, MMP-1, and MMP-13 [79,80]. The higher level of leptin in women may partially explain the greater frequency and severity of OA in postmenopausal women [6,81]. The role of adiponectin is dual. On one hand, it has anti-inflammatory properties, inducing M2 polarization of macrophages [82]. On the other hand, high levels of adiponectin in synovial fluid are associated with OA progression and the production of pro-inflammatory factors [6,58,83]. This dual role is context-dependent and influenced by the local cytokine milieu and adipokine balance, which can shift adiponectin activity toward either anti- or pro-inflammatory effects [81,84,85]. Resistin and visfatin also exhibit pro-inflammatory properties, inducing the production of IL-1β, TNF-α, and MMP [86,87].

The innate immune system initiates “sterile” inflammation in OA. The destruction of the ECM leads to the release of damage-associated molecular patterns (DAMPs), which are recognized by pattern recognition receptors (PRRs), primarily Toll-like receptors (TLRs) [88]. Fragments of the ECM, specifically low molecular weight hyaluronan, fragments of fibronectin, aggrecan, and type II collagen, are ligands for TLR-2 and TLR-4 [89]. Their binding to chondrocytes, macrophages, and FLS activates NF-κB and MAPK, enhancing the production of cytokines and proteases [90]. The HMGB1 protein, a nuclear protein released from necrotic or activated cells, binds to TLR-2, TLR-4, and RAGE, contributing to synovitis and cartilage degradation [91]. The heterodimer S100A8/A9 (calprotectin) is actively produced by macrophages and neutrophils, and its levels correlate with inflammation and destruction [92].

Pro-inflammatory stimuli exert their effects through a limited number of intracellular signaling cascades, which are key integrators of the signaling process [81]. The nuclear factor kappa-light-chain-enhancer of activated B cells (NF-κB) is considered a master regulator of inflammation in OA [6,7,12,81]. In a resting state, the NF-κB heterodimer (p50/p65) is bound to the inhibitory protein IκBα in the cytoplasm. Activation of IL-1β, TNF-α, or TLRs triggers a phosphorylation cascade through the IKK kinase complex, leading to the phosphorylation, ubiquitination, and degradation of IκBα. The released NF-κB translocates to the nucleus and activates the transcription of hundreds of genes, including those for pro-inflammatory cytokines (IL-6, TNF-α), chemokines, COX-2, iNOS, MMP-1, -3, -13, ADAMTS-4, -5 [6,93,94,95]. Hyperactivation of this pathway is one of the early and key events in the pathogenesis of OA. NF-κB is also involved in the formation of the SASP [96].

Another important intracellular signaling pathway is the MAPK family, which includes three main branches: ERK, JNK, and p38 MAPK. All of them are activated by stress stimuli and inflammatory cytokines [97,98,99]. p38 MAPK plays a key role in the production of pro-inflammatory cytokines and MMP-13 [97]. The c-Jun N-terminal kinase (JNK) phosphorylates and activates a component of the AP-1 complex (c-Jun). AP-1 (activator protein-1) synergistically with NF-κB activates genes, including MMP-13 [98]. ERK is generally associated with proliferation but is also activated by inflammatory stimuli and participates in the production of MMPs and PGE_2_ [99].

The JAK/STAT signaling pathway mediates the action of many cytokines, particularly those of the IL-6 family [93,100,101]. The binding of a cytokine to its receptor activates Janus kinases (JAK), which phosphorylate the receptor, creating docking sites for STAT proteins. Phosphorylated STATs (e.g., STAT3) dimerize and translocate to the nucleus, regulating the expression of genes involved in inflammation, cell survival, and MMP production [6,93]. For instance, the plant compound Epimedin C has demonstrated the ability to suppress ECM degradation in OA specifically by inhibiting STAT3 phosphorylation [102]. There are complex interactions between the STAT3 and NF-κB pathways, which can mutually enhance each other [103]. Currently, inhibitors of intracellular pathways such as JAK, p38 MAPK, and IKK are under development. Their systemic application is limited by the risk of side effects, necessitating the exploration and modification of the drug delivery route [104].

In addition to these primary pathways, a number of factors play a critical role in the pathogenesis of OA. Hypoxia-Inducible Factor-2α (HIF-2α) differs from the adaptive and protective HIF-1α; HIF-2α is a powerful catabolic regulator in OA [7,105]. Its expression is induced by NF-κB and other inflammatory signals, and it directly activates the transcription of MMP-13 and ADAMTS-4 genes, forming a positive feedback loop that exacerbates destruction [105]. Runt-related transcription factor 2 (RUNX2) serves as a key regulator of chondrocyte hypertrophy. In OA, pro-inflammatory cytokines increase RUNX2 activity, which stimulates the expression of MMP-13 and type X collagen, contributing to pathological hypertrophy and calcification of cartilage [49,106]. Early non-selective MMP inhibitors have been shown to cause severe side effects. Currently, efforts are underway to develop selective MMP-13 inhibitors [12].

One of the most significant achievements in recent years has been the recognition of the role of cellular senescence and the related phenomenon of inflammaging [107]. Cellular senescence is a state of irreversible cell cycle arrest in response to stressors (telomere shortening, oxidative stress, DNA damage). With age, the number of senescent cells accumulates, and a significant increase in the number of senescent chondrocytes has been detected in the affected cartilage of individuals with OA [107,108]. The major concern is the acquisition of a SASP-like secretory profile. Senescent chondrocytes with SASP begin to secrete a vast number of pro-inflammatory molecules: cytokines (IL-1β, IL-6, IL-8), chemokines, MMPs, and growth factors [96]. This secretion creates a pro-inflammatory microenvironment that disrupts the function of healthy cells, promotes ECM degradation, paracrinely induces senescence in neighboring cells, and recruits immune cells, further enhancing inflammation [108]. SASP factors act as key drivers of cartilage degeneration. They can diffuse into the subchondral bone, stimulating osteoclast activity and disrupting osteoblast function, leading to pathological bone remodeling (osteophytes, sclerosis) [108]. Thus, inflammaging serves as a link between aging and the development of chronic inflammation and joint degeneration. In turn, senolytics, drugs that selectively eliminate senescent cells, may represent a new strategy for OA therapy. The combination of dasatinib and quercetin has shown the ability to reduce the number of senescent chondrocytes and SASP in preclinical models, alleviating pain and protecting cartilage [108].

Progressive loss of chondrocyte integrity is one of the key features of OA. Chondrocyte death occurs through various pathways, each contributing to inflammation and degradation [49]. It is known that apoptosis is a classical form of programmed cell death [109,110]. Pro-inflammatory cytokines (IL-1β, TNF-α) are powerful inducers of chondrocyte apoptosis via both the extrinsic (death receptors) and intrinsic (mitochondrial) pathways [110,111]. Apoptosis leads to the release of cellular contents (DAMPs), thereby enhancing inflammation.

Pyroptosis is a highly pro-inflammatory form of cell death mediated by gasdermin D (GSDMD) and dependent on caspases-1/4/5/11 [112]. A key event is the activation of the NLRP3 inflammasome by various DAMPs (urate, ATP, cholesterol crystals) [113]. Activation of NLRP3 leads to the activation of caspase-1, which cleaves pro-IL-1β and pro-IL-18 into their active forms and cleaves GSDMD. The N-terminal fragment of GSDMD forms pores in the membrane, leading to cell lysis and massive release of active IL-1β and IL-18, causing a powerful inflammatory response. It should be noted that pyroptosis can proceed via canonical and non-canonical inflammasome pathways, involving caspase-1 and caspases-4/5 in humans or their murine homolog caspase-11, respectively [114,115]. Given that many OA studies rely on murine models, the dominant role of caspase-11 should be considered when interpreting and translating these findings to human biology. Pyroptosis has been detected in chondrocytes and synovial cells in OA, and its inhibition reduces disease severity in models [116,117].

Ferroptosis is a recently discovered form of regulated cell death characterized by the accumulation of iron-dependent lipid peroxides [118]. The central regulator is the enzyme glutathione peroxidase 4 (GPX4), which neutralizes lipid hydroperoxides. Suppression of GPX4, depletion of glutathione (GSH), or disruption of the cystine/glutamate antiporter system (system Xc-) leads to the accumulation of reactive oxygen species (ROS) and membrane lipid peroxidation, resulting in cell death [119]. There is increased iron content in the cartilage and synovial fluid of OA patients [120]. Mechanical overload and inflammatory cytokines suppress GPX4 expression in chondrocytes, rendering them susceptible to ferroptosis [121]. Induction of ferroptosis leads to degradation of type II collagen and cell death [122]. In preclinical studies, ferroptosis inhibitors (ferrostatin-1) [108,122,123] and pyroptosis inhibitors (NLRP3 inhibitors, such as CY-09) [116,124] effectively slow OA progression. The development of nanomaterials with enzyme-like activity (nanoenzymes), for example, based on iron atoms (Fe SAzymes) that mimic antioxidant enzymes (SOD, CAT, GPX) and suppress ferroptosis, is a promising approach [8]. Fe SAzymes are nanoscale catalytic systems based on single iron atoms capable of modulating redox processes and inhibiting ferroptosis. In preclinical models, functionalized Fe SAzymes (e.g., conjugated with cartilage-targeting peptides and siRNA against MMP13) have been shown to reduce matrix degradation, increase GPX4 expression, restore mitochondrial function, and suppress inflammation in cartilage tissue, likely through modulation of glutathione metabolism and pro-inflammatory signaling pathways, including IL-17 [8].

One of the links in the pathogenesis of OA is mitochondrial dysfunction and oxidative stress. Mitochondria in chondrocytes during OA are functionally impaired: the activity of respiratory complexes, ATP synthesis, and membrane potential are reduced [125,126]. Dysfunctional mitochondria become a major source of ROS, leading to oxidative stress, activation of MAPK and NF-κB, and induction of apoptosis and ferroptosis [127,128]. Mitochondrial DNA (mtDNA), released from damaged mitochondria, is itself a potent DAMP and can activate TLR9 and the NLRP3 inflammasome, creating a vicious cycle of inflammation [129].

The study by Ma et al. (2025) demonstrated a fundamentally new mechanism linking cholesterol metabolism and inflammation [12]. The authors showed that osteocytes in the subchondral bone increase cholesterol uptake and transfer their mitochondria to chondrocytes during OA. The accumulation of foreign mtDNA in the cytosol of chondrocytes is recognized as a danger signal, activating the pro-inflammatory cGAS-STING pathway. This finding highlights the importance of intercellular transfer of organelles and metabolic crosstalk between joint tissues. Moreover, dampening mitochondrial transfer could serve as a new therapeutic approach for OA [12].

Dysregulation of lipid metabolism has long been associated with OA. A study conducted in 2025 showed that cold exposure exacerbates the course of OA in mice by suppressing the expression of apolipoprotein E (APOE) in chondrocytes [130]. APOE plays a key role in the export of lipids from cells. Its deficiency leads to lipid accumulation, increased levels of ROS, mitochondrial dysfunction, and apoptosis. Activation of the LXRβ receptor restored APOE levels and mitigated the negative effects of cold [130]. This research clarifies the exacerbation of pain in cold weather and opens up new therapeutic targets related to lipid metabolism, such as acting on LXR receptors to correct lipid metabolism and restore APOE levels [130]. Accumulation of lipids and increased oxidative stress not only contribute to cartilage degradation but may also be involved in pain generation. Elevated ROS and lipid mediators can enhance the production of pro-inflammatory cytokines and sensitize nociceptive nerve endings within the joint, promoting neuroinflammation [131].

The ultimate result of the activation of inflammatory and catabolic pathways is the irreversible destruction of the ECM of cartilage. MMPs and aggrecanases (ADAMTS) are a family of zinc-dependent endopeptidases produced by chondrocytes and synoviocytes in response to inflammatory stimuli [132,133]. Collagenases (MMP-1, MMP-8, MMP-13) degrade fibrillar collagens. MMP-13 has the highest specificity for collagen type II and is a key enzyme in the irreversible degradation of cartilage in OA [6,50,134]. Its expression is regulated by the NF-κB and MAPK pathways, as well as by RUNX2 and HIF-2α factors. Stromelysins (MMP-3) degrade proteoglycans, fibronectin, and play a role in the activation of other MMPs [135]. Gelatinases (MMP-2, MMP-9) degrade denatured collagen and are involved in angiogenesis [136]. Aggrecanases (ADAMTS-4, ADAMTS-5) are the primary enzymes that degrade aggrecan [137]. Tissue inhibitors of metalloproteinases (TIMPs) normally restrain the activity of MMPs (TIMP-1, -2, -3, -4). However, in OA, this balance is disrupted: the production of MMPs significantly exceeds the level of TIMPs, leading to net degradation of the ECM [138]. TIMP-3 is unique in that it inhibits not only MMPs but also ADAMTS [139]. Overall, the inhibition of matrix-degrading enzymes shows great potential as a future therapeutic strategy for the treatment of OA [140,141].

## 3. Selection of Cell Type for the Development of Products for Inflammatory Joint Disease Therapy

### 3.1. MSCs

MSCs have been widely studied as a therapeutic agent for OA in both pre-clinical and clinical settings. MSC-based therapies have proven safe and shown potential efficacy in pain relief, slowing cartilage degeneration, and, in some cases, promoting cartilage repair [142]. While initial hopes centered on their chondrogenic differentiation and direct tissue replacement capacity, evidence has increasingly highlighted their immunomodulatory potential. This shift is supported by the observed lack of a clear dose–response relationship for intra-articularly administered autologous MSCs [143]. For example, significant pain reduction and higher functional scores have been reported with the intra-articular injection of 2 million MSCs compared to the injection of 10 million or 50 million MSCs [144]. Overall, research investigating the correlation between injected cell number and clinical outcomes has produced inconsistent results regarding the optimal [145,146].

This variability is strongly influenced by MSC heterogeneity. Cells from different tissue sources exhibit distinct functional profiles. Adipose-derived MSCs often show stronger immunosuppressive effects, whereas bone marrow-derived MSCs may better support angiogenesis and interaction with immune cells. Donor-related factors such as age and health status affect cell potency: MSCs from older donors demonstrate increased senescence, reduced proliferation, and impaired differentiation capacity. Culture conditions further modulate MSC function hypoxia enhances survival, angiogenic potential, and secretion of regenerative factors, while prolonged passaging leads to loss of potency, clonal selection, and increased senescence [45]. Additionally, heterogeneity at the subpopulation level results in cells with distinct transcriptomic and secretory profiles, directly influencing their immunomodulatory and regenerative activity [147]. Differences in transcriptomic and secretomic profiles, which underlying MSC subpopulation heterogeneity and lead to variability in cytokine and extracellular vesicle production, thereby altering immunomodulatory and regenerative effects. Functionally, these subpopulations exhibit divergent capacities for chondrogenic differentiation, immune suppression and meaning that therapeutic outcomes depend on their relative composition [148].

The predominance of paracrine mechanisms is further evidenced by a recurring discrepancy: while many studies report short-term symptomatic benefits from MSC therapy, long-term structural efficacy remains unproven [149]. Imaging data confirm poor defect filling post-treatment [150]. For instance, a study of a single intra-articular injection of adipose-derived MSCs in knee OA found no pronounced tissue regeneration, yet reported maintained joint function, reduced pain, and stabilization of the lesion size for six months compared to controls [151]. Thus, the modest long-term effectiveness of MSC-based products may stem not only from their limited direct engraftment potential but also from suboptimal dosing, delivery protocols, and therapeutic combinations [152].

The primary therapeutic mechanism of MSCs in OA is now attributed to their potent immunomodulatory and anti-inflammatory impact on the joint milieu. Recent evidence shows that tissue-engineered cartilage derived from MSCs can induce a shift in resident macrophages towards the anti-inflammatory M2 phenotype, characterized by elevated IL-10 and reduced IL-1β, resulting in superior construct viability compared to autologous chondrocytes or BM-MSC co-transplants [153]. MSCs dampen innate immunity by reducing NK cell activity and promoting M2 polarization [154]. They also modulate adaptive immunity, as intra-articular administration suppresses B- and T-cell proliferation/activation, inhibits Th17 cells, and may expand regulatory Treg and Breg populations [155]. Furthermore, MSCs exert a direct immunosuppressive effect on mast cells, with bone marrow–derived mesenchymal stem cells shown to inhibit degranulation, pro-inflammatory cytokine release, and chemotaxis in experimental models [156].

Accumulating evidence indicates that the therapeutic efficacy of MSCs is largely mediated by their secretome, specifically exosomes and other extracellular vesicles, rather than the cells per se. Recent studies demonstrate that MSC-derived exosomes exhibit immunomodulatory capabilities comparable to, and in some contexts superior to, their cellular counterparts. For instance, in models of inflammatory arthritis, these exosomes suppress in vivo inflammation more potently than intact MSCs [157]. Mechanistically, exosomes from adipose-derived stromal cells (ASCs) have been shown to downregulate pro-inflammatory mediators (IL-6, NFκB, TNF-α) and upregulate anti-inflammatory IL-10 in activated synovial fibroblasts [158]. Bioinformatics analysis further suggests that ASCs primed with native OA synovial fluid can orchestrate an anti-inflammatory shift in the joint environment by polarizing resident macrophages to an M2 phenotype, inhibiting T-cell proliferation, promoting a Th1-to-Th2 switch, and expanding Treg populations—all contributing to cartilage repair [159]. Supporting this, co-culture of ASCs with chondrocytes in OA synovial fluid reduces chondrocyte expression of the catabolic enzyme MMP13 and elevates levels of anti-inflammatory cytokines (IL-1RA, IL-10, IL-13) [160].

In another study, extracellular vesicles from MSCs increased chondrocyte proliferation and ECM synthesis by inhibiting M1 macrophage infiltration and promoting M2 macrophage polarization, while simultaneously reducing the expression of inflammatory cytokines IL-1β and TNF-α [161], as well as suppressing NK cell activation [162,163], which may potentially contribute to cartilage repair. Thus, the obtained data confirm the mediated regenerative and immunomodulatory properties of MSCs upon interaction with the intra-articular environment in OA patients.

### 3.2. Macrophages

It is known that macrophages play a central role in the development of chronic inflammation, pain, cartilage destruction, and bone remodeling in OA. However, macrophages are also involved in tissue repair, including cartilage [164]. The macrophage population is heterogeneous and dynamic, with phenotypic transitions induced by various stimuli [15]. To effectively leverage the transition of macrophages to an anti-inflammatory phenotype for the treatment of OA, it is essential to understand the heterogeneity of the macrophage population and their interactions with surrounding cells and tissues in the joint. To date, few clinical studies have been conducted on macrophage polarization in OA [165,166]. Most evidence is derived from in vitro studies, highlighting the novelty of this therapeutic target, though its clinical potential is recognized [15]. Due to their ability to change and adapt to various exogenous and endogenous factors, macrophages can rapidly change their functional profile through a process known as polarization [167].

Macrophage polarization from the pro-inflammatory M1 to the anti-inflammatory M2 phenotype is mediated by a range of specific factors, including: phagocytosis of damaged cells at the site of inflammation; cytokines; lipid mediators such as eicosapentaenoic and docosahexaenoic acids; microRNAs; and long non-coding RNAs. When a macrophage transitions from the M1 to the M2 phenotype, the expression of many genes changes [168,169]. M2 macrophages play a central role in the transition from the inflammatory to the proliferative phase of tissue repair. In doing so, macrophages “clean up” the damaged tissue, reconstructing the ECM. Moreover, macrophages play a key role in the biosynthesis of specialized pro-resolving mediators (SPMs) [170]. SPMs possess anti-inflammatory capacity, as well as regenerative and analgesic properties [171]. M2 macrophages express large amounts of arginase-1 (ARG-1), secrete platelet-derived growth factor (PDGF); transforming growth factor (TGF); vascular endothelial growth factor (VEGF); the anti-inflammatory cytokines IL-10 and IL-1RA; chemokine ligand 18 (CCL18); and insulin-like growth factor (IGF). These factors contribute to creating a pro-chondrogenic environment, promoting chondrocyte survival, resolving inflammation, and facilitating tissue repair [172,173]. Currently, the plasticity of the macrophage phenotype is actively being studied, with macrophages conventionally classified into four M2 subtypes: M2a, M2b, M2c, and M2d [174]. However, the effect of “phenotypic drift” can lead to a change in the anti-inflammatory phenotype of cells to a pro-inflammatory one under the influence of cytokine signals [167], which imposes certain limitations and requires further research. The literature describes an experiment involving the “locking” of macrophages in the M2a phenotype by simultaneously knocking out TNFR1 and overexpressing IL-4 using CRISPR-Cas9 gene editing technology [175]. However, the question of the effectiveness, safety, and potential for the translation of this technology into medicine remains unresolved.

A key limitation of M2 macrophage-based approaches is their phenotypic instability. Current strategies to stabilize the M2 phenotype are primarily based on maintaining a supportive microenvironment, including sustained exposure to IL-4/IL-13–STAT6 signaling and activation of PPARγ-dependent metabolic programs that promote oxidative phosphorylation and fatty acid oxidation, which are characteristic of M2 polarization. In addition, epigenetic regulation (e.g., DNA methylation, histone modifications, and non-coding RNAs) contributes to the stabilization of macrophage phenotypes, providing a potential mechanism for maintaining anti-inflammatory states [176,177]. Biomaterial-based approaches can further support M2 polarization by creating a spatiotemporally controlled microenvironment that first promotes M1→M2 transition and then maintains regenerative signaling through controlled release of immunomodulatory factors [177]. Importantly, crosstalk with mesenchymal stem cells may additionally stabilize the M2 phenotype, as MSC-derived factors (e.g., TGF-β, PGE2) suppress inflammation and promote sustained immunoregulatory activity.

Therefore, we propose that polarized M2 macrophages obtained, for example, from peripheral blood monocytes of the patient or from a hypoimmunogenic line of allogenic iPSCs, and MSCs are key candidate components for tissue-engineered cartilage grafts, where their anti-inflammatory properties can boost regeneration and limit adverse immune responses post-implantation. Extending this rationale, M2 macrophage-derived exosomes may offer a subsequent, cell-free therapeutic strategy building on the promise of MSC exosomes [162].

### 3.3. iPSCs

The advent of iPSCs technology revolutionized stem cell research by bypassing the ethical issues of embryonic stem cells and accelerating progress in cell therapy [178]. Advances in maintaining pluripotent iPSCs and differentiating them into specific lineages have opened a new era for regenerative medicine, enabling the development of replacement tissues [179]. Numerous clinical trials are now evaluating iPSC-derived cells for conditions including myocardial infarction [180,181,182], spinal cord injury [183], Parkinson’s disease [184,185], and age-related macular degeneration [186,187,188,189,190]. iPSCs are also being leveraged to generate immune cells (e.g., NK cells, T-lymphocytes) for cancer immunotherapy [191,192] and to address immunological disorders and thrombocytopenia [193]. Notably, iPSC-derived MSCs are under investigation for knee osteoarthritis in a Phase I trial [20]. While safety concerns have limited the global number of iPSC clinical trials, long-term studies (e.g., 10-year follow-up of transplanted oligodendrocyte precursors, albeit ESC-derived) report no tumorigenicity [194,195].

**Figure 2:**
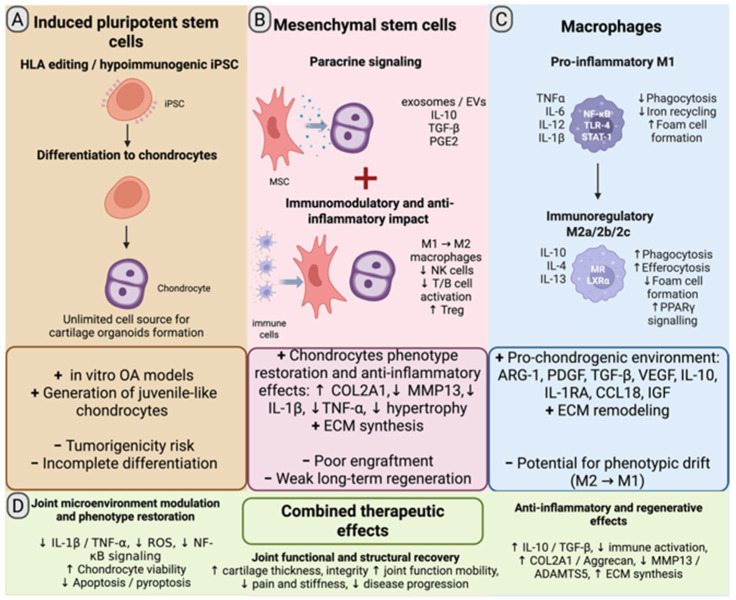
Integrated cell-based strategies for osteoarthritis therapy: synergistic roles of iPSCs, MSCs, and macrophages. (**A**) iPSCs provide a scalable source of juvenile-like chondrocytes and cartilage organoids, including hypoimmunogenic lines; limitations include residual undifferentiated cells and incomplete maturation. (**B**) MSCs mediate paracrine immunomodulation via cytokines and extracellular vesicles, promoting ECM synthesis and suppression of inflammation, with limited long-term engraftment. (**C**) Macrophages regulate inflammation through M1–M2 polarization, supporting ECM remodeling and a pro-chondrogenic microenvironment, but exhibit phenotypic instability. (**D**) Combined cell-based approaches reduce inflammatory signaling, oxidative stress, and regulated cell death, enhance chondrocyte viability and ECM production, and promote structural and functional restoration of cartilage. Figure created by the authors. Created in BioRender. Ruchko, E. (2026) https://BioRender.com/v45eb5u.

iPSCs could potentially serve as an alternative source of chondrocytes [35,44] (Figure 2). A case of successful transplantation of cartilage organoids obtained from iPSCs of a macaque was published, which were implanted into a cartilage defect in the knee joint of primates. As a result of this experimental study, tissue regeneration was achieved that persisted for at least 4 months without any occurrence of immune rejection. This research demonstrated the possibility of remodeling articular cartilage in primates, laying the groundwork for developing treatment methods for cartilage defects based on allogeneic pluripotent stem cells [33].

Reprogramming somatic cells into iPSCs is a resource-intensive process, demanding substantial time and effort for clone selection, characterization, and quality control. Consequently, therapies based on patient-specific (autologous) iPSCs are costly and unsuitable for acute conditions. A promising alternative involves creating banks of pre-characterized, universally compatible iPSC lines. This can be achieved by developing homozygous lines or by using gene editing (e.g., HLA knockout) to generate immunotolerant iPSCs [196,197,198,199]. While these strategies could provide readily available “off-the-shelf” cell products, the underlying technologies are still under development and require comprehensive safety validation.

## 4. Complex 3D Cellular Constructs

Research on complex multi-component 3D cellular constructs, termed assembloids or organoids, has grown rapidly in recent years. Organoids are self-organizing 3D in vitro cultures that recapitulate certain organ functions through their heterogeneous cell composition [200]. Assembloids represent a more advanced model, formed either by integrating distinct organoids or by co-differentiating multiple specialized cell types from the outset, often derived from stem cells. While significant progress has been made with neural assembloids for studying development and disease [201], these systems have broad utility in modeling tissue-level interactions and pathophysiology. Recent advances in the automation of large-scale production and phenotyping of organoids [202,203] will also contribute to identifying structural features and cell–cell interactions. For example, the possibility of modeling embryo implantation using endometrial assembloids has been demonstrated [204,205], as well as the behavior of intestinal stem cells in gastrointestinal assembloids [206]. The use of iPSCs to create such complex organoids is the most frequent case; and, such developments are currently used only for scientific purposes. Similar developments for the treatment of inflammatory joint diseases do not exist. Furthermore, in the authors’ opinion, most publications on organoids describe their application for disease modeling or for studying therapeutic agents, rather than as an independent means of regenerative medicine [207]. It is important to note that organoids, including cartilage organoids, represent not just a simple summation of several components, but rather an organic interaction and dynamic organization among various components [208], which requires further study.

**Figure 3:**
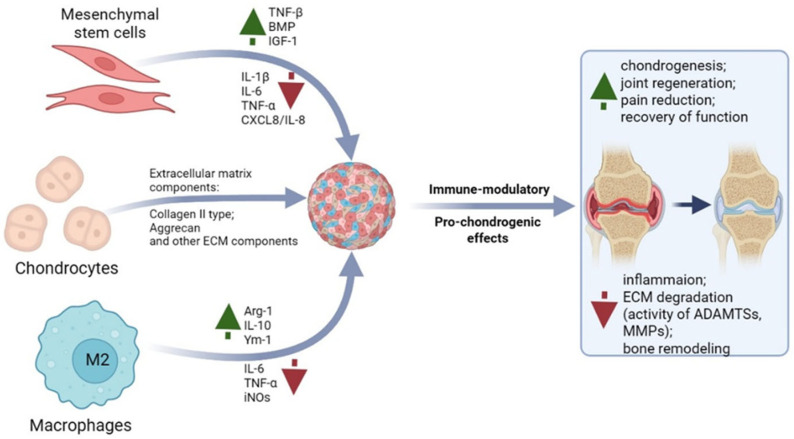
Proposed Mechanism of Action of a Multicomponent Spheroid with Anti-Inflammatory Properties. Figure created by the authors. Created in BioRender. Голубинская, П. (2026) https://BioRender.com/v9ocl1v.

Recent research is exploring multi-cellular approaches for OA therapy (Figure 3). Key findings suggest that macrophage polarization towards an M2 phenotype can inhibit OA-characteristic chondrocyte hypertrophy [209]. Furthermore, while MSCs alone reduce general inflammation, only their co-culture with macrophages effectively reverses the inflammatory chondrocyte phenotype associated with OA [210]. Maintaining chondrocyte homeostasis is highly dependent on culture conditions, as serial passaging induces a pro-inflammatory shift, including increased IL-6 expression [211]. Therefore, based on this evidence, supplementing chondrocyte monocultures with MSCs presents a promising strategy to mitigate their dedifferentiation and adverse phenotypic changes [212].

In vitro studies highlight the significant therapeutic promise of chondrocyte-MSC co-culture, evidenced by enhanced ECM synthesis [213,214]. This effect is largely attributed to MSC paracrine signaling. The co-culture improves chondrogenesis and exerts anti-inflammatory and regenerative effects, primarily mediated by extracellular vesicles (EVs) [215]. In fact, EVs harvested from such co-cultures are being explored as a novel OA therapeutic strategy [215]. Furthermore, various two-dimensional and three-dimensional, static and dynamic co-culture systems of MSCs and chondrocytes are being investigated to obtain therapeutic extracellular vesicles [216].

The tissue origin of MSCs can be another critical factor. In a minipig model, iPSC-derived MSCs demonstrated superior therapeutic efficacy compared to implants based on bone marrow-derived MSC derivatives [217]. The iPSC-MSC group not only showed better clinical outcomes but also a more favorable molecular profile: higher expression of the cartilage-specific collagen COL2A1 and minimal expression of fibrosis-associated (COL1A1) and hypertrophy-associated (COL10A1) markers [217].

## 5. Conclusions

The development of effective OA treatment is being actively pursued through regenerative medicine strategies centered on multi-component cellular implants. The core therapeutic agent may consist of chondrocytes (sourced directly or differentiated from MSCs/iPSCs), supplemented with immunomodulatory MSCs and strategically polarized M2 macrophages to create an anti-inflammatory, pro-regenerative niche. While this integrated approach holds considerable translational potential, its clinical realization necessitates systematic preclinical and clinical validation. Key research priorities include long-term safety assessment and the engineering of optimized delivery matrices. Success in these endeavors could position advanced multi-cellular products as a compelling alternative to invasive joint replacement surgery, offering a more biological solution for OA management.

## Data Availability

No new data were created or analyzed in this study.

## Abbreviations

The following abbreviations are used in this manuscript:

MSCs	Mesenchymal stem cells
HSCs	Hematopoietic stem cells
SVF	Stromal vascular fraction
OA	Osteoarthritis
SASP	Senescence-associated secretory phenotype
ACI	Autologous chondrocyte implantation
ECM	Extracellular matrix
iPSCs	Induced pluripotent stem cells
MMPs	Matrix metalloproteinases
NO	Nitric oxide
sIL-6R	Soluble receptor sIL-6R
DAMPs	Damage-associated molecular patterns
PRRs	Pattern recognition receptors
TLRs	Toll-like receptors
NF-κB	The nuclear factor kappa-light-chain-enhancer of activated B cells
JNK	c-Jun N-terminal kinase
HIF-2α	Hypoxia-Inducible Factor-2α
RUNX2	Runt-related transcription factor 2
GSDMD	Gasdermin D
ROS	Reactive oxygen species
TIMPs	Tissue inhibitors of metalloproteinases
APOE	Apolipoprotein E
ASCs	Adipose-derived stromal cells
SPMs	Specialized pro-resolving mediators
ARG-1	Arginase-1
PDGF	Platelet-derived growth factor
TGF	Transforming growth factor
VEGF	Vascular endothelial growth factor
CCL18	Chemokine ligand 18
IGF	Insulin-like growth factor
EVs	Extracellular vesicles
